# Deletion of CD2-Like (CD2v) and C-Type Lectin-Like (EP153R) Genes from African Swine Fever Virus Georgia-∆9GL Abrogates Its Effectiveness as an Experimental Vaccine

**DOI:** 10.3390/v12101185

**Published:** 2020-10-20

**Authors:** Douglas P. Gladue, Vivian O’Donnell, Elizabeth Ramirez-Medina, Ayushi Rai, Sarah Pruitt, Elizabeth A. Vuono, Ediane Silva, Lauro Velazquez-Salinas, Manuel V. Borca

**Affiliations:** 1 Plum Island Animal Disease Center, ARS, USDA, Greenport, NY 11944, USA; vivian.Odonnell@usda.gov (V.O.); Elizabeth.Ramirez@usda.gov (E.R.-M.); Ayushi.Rai@usda.gov (A.R.); Sarah.Pruitt@usda.gov (S.P.); Elizabeth.Vuono@usda.gov (E.A.V.); Ediane.Silva@usda.gov (E.S.); Lauro.Velazquez@usda.gov (L.V.-S.); 2 Plum Island Animal Disease Center, APHIS, USDA, Greenport, NY 11944, USA; 3 Department of Anatomy and Physiology, Kansas State University, Manhattan, KS 66506, USA; 4 Oak Ridge Institute for Science and Education (ORISE), Oak Ridge, TN 37830, USA; 5 Department of Pathobiology and Population Medicine, Mississippi State University, Mississippi State, MS 39762, USA

**Keywords:** ASFV, ASF, recombinant viruses, vaccine, CD2, EP402R, EP13R, lectin

## Abstract

African swine fever virus (ASFV) is currently the most dreaded infectious disease affecting the global swine production industry. There is no commercial vaccine available, making the culling of infected animals the current solution to control outbreaks. Effective experimental vaccines have been developed by deleting virus genes associated with virulence. Deletion of the ASFV 9GL gene (∆9GL) has resulted in the attenuation of different ASFV strains, although the degree of attenuation varies across isolates. Here, we investigated the possibility of the increased safety of the experimental vaccine strain ASFV-G-Δ9GL by deleting two additional virus genes involved in pathogenesis, CD2v, a CD2 like viral encoded gene from the EP402R open reading frame (ORF), and C-type lectin-like viral gene, encoded from the EP153R ORF. Two new recombinant viruses were developed, ASFV-G-Δ9GL/ΔCD2v and ASFV-G-Δ9GL/ΔCD2v/ΔEP153R, harboring two and three gene deletions, respectively. ASFV-G-Δ9GL/ΔCD2v/ΔEP153R, but not ASFV-G-Δ9GL/ΔCD2v, had a decreased ability to replicate in vitro in swine macrophage cultures when compared with parental ASFV-G-Δ9GL. Importantly, ASFV-G-Δ9GL/ΔCD2v and ASFV-G-Δ9GL/ΔCD2v/ΔEP153R induced almost undetectable viremia levels when inoculated into domestic pigs and failed to protect them against challenge with parental virulent ASFV-Georgia, while ASFV-G-Δ9GL offered robust protection during challenge. Therefore, the deletion of CD2-like and C-type lectin-like genes significantly decreased the protective potential of ASFV-G-Δ9GL as a vaccine candidate. This study constitutes an example of the unpredictability of genetic manipulation involving the simultaneous deletion of multiple genes from the ASFV genome.

## 1. Introduction

African swine fever virus (ASFV), the only member of the Asfarviridae family, has a 180–190 kilobase double-stranded DNA genome that encodes for more than 150 open reading frames (ORFs) and is the etiological agent of African swine fever (ASF), a highly dreaded disease of swine [1]. Historically restricted to sub-Saharan African countries and Sardinia (Italy), an outbreak occurred in 2007 in the Caucasus region, rapidly spreading to neighboring countries in central Europe, China and throughout Asia. The ASFV strain causing the pandemic is highly contagious and lethal for domestic pigs. The disease is currently considered the most significant threat to the global swine production industry and available consumable worldwide protein [2]. No commercial vaccines are available to prevent and/or control ASF, making the quarantine and culling of infected animals the primary responses to outbreak containment.

Experimental vaccines demonstrating protection against the current pandemic ASF virus strain use recombinant live attenuated viruses derived from the Georgia 2007 isolate [3,4,5]. These recombinant viruses are attenuated after the deletion of one or more genes from the field isolate genome using established genetic manipulation strategies. We previously developed several ASFV vaccine candidates by deleting the I177L, 9GL, and MGF genes [3,4,5]. Some of these viruses, although effective, require an enhanced safety profile [4,5]. The inclusion of additional gene deletions to achieve this goal has produced diverse results depending on the cumulative deletions [6,7]. Understanding the role of individual genes in virus virulence, virus replication, and immunogenicity is critical for developing improved vaccines, and understanding how different combinations of deletions can affect vaccine platforms is crucial for next-generation live-attenuated vaccines.

The deletion of 9GL, a highly conserved gene across ASFV strains, produced different attenuation profiles depending on the strain considered. While Malawi [8] and Pret4 [9] isolates were completely attenuated following the deletion of 9GL, the Georgia2010 isolate (ASFV-G-Δ9GL) remained virulent when inoculated at high doses [5]. Attempts to increase the attenuation of ASFV-G-Δ9GL by including the same deletion producing attenuation in the ASFV-G-ΔMGF virus resulted in a significant reduction in a protective effect of the resulting virus, ASFV-G-Δ9GL/ΔMGF [7]. In contrast, the deletion of the UK gene, which by itself does not induce a significant phenotypic change within the ASFV Georgia2010 isolate [10], drastically increased the attenuation of ASFV-G-Δ9GL without affecting the protective efficacy of the resulting virus, ASFV-G-Δ9GL/ΔUK [6]. It has been recently reported that the deletion of the CD2-like (CD2v) gene from the Georgia isolate, a deletion that does not result in attenuation even at low doses by the intranasal route [11], increased the attenuation of a recombinant Georgia derivative virus harboring a deletion similar to that of ASFV-G-ΔMGF [12]. Experimentally evaluating the effects of different gene deletion combinations is critical for identifying effective vaccine candidates. 

Here, we investigated enhancing the safety profile of ASFV-G-Δ9GL by deleting two additional genes involved in different aspects of virus pathogenesis, CD2-like (CD2v, first identified as 8DR) and Lectin-like (EP153R, first identified as 8CR) genes. Recombinant viruses were developed harboring two or three gene deletions (ASFV-G-Δ9GL/ΔCD2v and ASFV-G-Δ9GL/ΔCD2v/ΔEP153R, respectively) that were then tested for their ability to replicate in vitro and in vivo. Results indicate that these deletions significantly decreased the ability of parental ASFV-G-Δ9GL to replicate in vitro and to protect domestic swine against challenge with virulent virus. While the deletion of CD2v and EP153R genes may increase the safety profile of ASFV-G-Δ9GL, the deletions also reduce its protective efficacy as a vaccine candidate. This outcome emphasizes the need to experimentally evaluate the effect of individual and cumulative gene deletions when studying modified viruses as potential vaccine candidates.

## 2. Materials and Methods

### 2.1. Viruses and Cells 

Primary swine macrophage cell cultures were prepared from defibrinated swine blood as previously described [13]. Briefly, heparin-treated swine blood was incubated at 37 °C for 1 h to allow the sedimentation of the erythrocyte fraction. Mononuclear leukocytes were separated by flotation over a Ficoll-Paque (Pharmacia, Piscataway, NJ, USA) density gradient (specific gravity, 1.079). The monocyte/macrophage cell fraction was cultured in plastic Primaria (Falcon; Becton Dickinson Labware, Franklin Lakes, NJ, USA) tissue culture flasks containing macrophage media, composed of RPMI 1640 Medium (Life Technologies, Grand Island, NY, USA) with 30% L929 supernatant and 20% fetal bovine serum (HI-FBS, Thermo Scientific, Waltham, MA, USA) for 48 h at 37 °C under 5% CO_2_. Adherent cells were detached from the plastic by using 10 mM EDTA in phosphate buffered saline (PBS) and were then reseeded into Primaria T25, 6- or 96-well dishes at a density of 5 × 10^6^ cells per ml for use in assays 24 h later. ASFV Georgia (ASFV-G) was a field isolate kindly provided by Dr. Nino Vepkhvadze, from the Laboratory of the Ministry of Agriculture (LMA) in Tbilisi, Republic of Georgia [4].

Comparative growth curves between parental ASFV-G, ASFV-G-Δ9GL, ASFV-G-Δ9GL/ΔCD2v and ASFV-G-Δ9GL/ΔCD2v/ΔEP153R were performed in primary swine macrophage cell cultures. Preformed monolayers were prepared in 24-well plates and infected at a MOI (Multiplicity of Infection) of 0.1 (based on tissue culture infectious doses (TCID_50_) previously determined in primary swine macrophage cell cultures). After 1 h of adsorption at 37 °C under 5% CO_2_ the inoculum was removed, and the cells were rinsed two times with PBS. The monolayers were then rinsed with macrophage media and incubated for 2, 24, 48, 72 and 96 h at 37 °C under 5% CO_2_. At appropriate times post-infection, the cells were frozen at ≤−70 °C and the thawed lysates were used to determine the titers by TCID_50_/mL in primary swine macrophage cell cultures. All samples were run simultaneously to avoid inter-assay variability. Virus titration was performed on primary swine macrophage cell cultures in 96-well plates. Virus dilutions and cultures were performed using a macrophage medium. Presence of the virus was assessed by the presence of the cytopathogenic effect (confirmed by hemadsorption in the case of ASFV-G, ASFV-G-Δ9GL and fluorescence in the case of ASFV-G-Δ9GL/ΔCD2v and ASFV-G-Δ9GL/ΔCD2v/ΔEP153R. Virus titers were calculated as previously described [14].

### 2.2. Construction of the Recombinant Viruses

**Figure 1:**
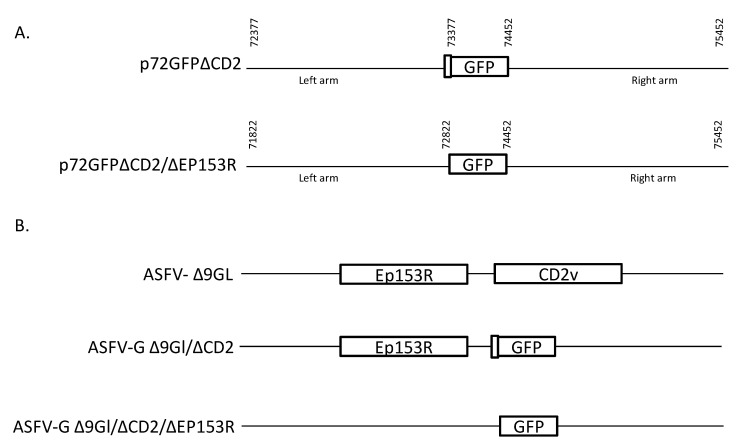
Schematic for the development of ASFV-G-Δ9GL/ΔCD2v and ASFV-G-Δ9GL/ΔCD2v/ΔEP153R. (**A**) The transfer vectors contain the p72 promoter and a GFP cassette with flanking arms. As indicated transfer vector p72GFP∆CD2 has a residual 9 nucleotides of CD2 before the GFP indicated by the empty box; the flanking left and right arms positions are indicated and were designed to have flanking ends to both sides of the deletion/insertion cassette. (**B**) The resulting recombinants ASFV-G-Δ9GL/ΔCD2v and ASFV-G-Δ9GL/ΔCD2v/ΔEP153R with the cassette inserted are shown on the bottom.

Recombinant ASFV-G-Δ9GL/ΔCD2v and ASFV-G-Δ9GL/ΔCD2v/ΔEP153R were generated by homologous recombination using parental virus ASFV-G-Δ9GL and recombination transfer vectors p72GFPΔCD2v and p72GFPΔCD2v/ΔEP153R following previously described procedures [6]. The left flanking genomic region between ASFV genome positions 72377–73377 for p72GFPΔCD2v and 71822 and 72822 for p72GFPΔCD2v/ΔEP153R, while the right arms for both vectors are the same between genome positions 74452 and 75452. Both transfer vectors harbor a reporter gene cassette containing the green fluorescent protein (GFP) gene under the control of the ASFV p72 late gene promoter as previously described [15]. Recombinant transfer vectors were obtained by DNA synthesis (Epoch Life Sciences, Sugar Land, TX, USA). This construction created a 1073-nucleotide deletion between nucleotide positions 73378–74451 in the case of ASFV-G-Δ9GL/ΔCD2v and a 1629-nucleotide deletion between nucleotide positions 72823–74451 in the case of ASFV-G-Δ9GL/ΔCD2v/ΔEP153R (Figure 1). These constructs produce the deletions in CD2v (leaving only the first 9 nucleotides in CD2) for ASFV-G-Δ9GL/ΔCD2v and completely deleting CD2 and EP152R genes in ASFV-G-Δ9GL/ΔCD2v/ΔEP153R. Macrophage cell cultures were infected with ASFV-G-Δ9GL and transfected with either p72GFPΔCD2v or p72GFPΔCD2v/ΔEP153R. CD2v recombinant viruses were purified to homogeneity by successive rounds of limiting dilution purification. ASFV DNA was extracted from infected cells and full-length sequence using next generation sequencing (NGS) was performed as described previously [4] using an Ion PGM sequencer and standard sequencing protocols. The analysis of the sequence was done using CLC Genomics Workbench software version 20 (QIAGEN, Hilden, Germany).

### 2.3. Detection of Anti-ASFV Antibodies

Anti-ASFV antibodies in the sera of infected animals were quantified using an in-house developed ELISA as previously described [9]. Briefly, ASFV was amplified in Vero cells [16], infected cells were then treated with Tween 80 (G-Biosciences, Saint Louis, MO, USA) and sodium deoxycholate (Sigma, Saint Louis, MO, USA) to a final concentration of 1% (*v/v*) and cell antigens were stored at ≤−70 °C. Maxisorb ELISA plates (Nunc, Saint Louis, MO, USA) were coated with 1 µg per well of either infected cell or uninfected cell antigen and blocked with phosphate buffered saline containing 10% skim milk (Merck, Kenilworth, NJ, USA) and 5% normal goat serum (Sigma). Swine sera were tested at multiple dilutions against both infected and uninfected cell antigen, with reactivity detected by an anti-swine IgG–horseradish peroxidase conjugate (KPL, Gaithersburg, MD, USA) and SureBlue Reserve peroxidase substrate (KPL). Plates were read at OD630 in an ELx808 plate reader (BioTek, Shoreline, WA, USA). Swine sera were considered positive for ASFV-specific antibodies if the OD630 ratio of the reaction against infected cell antigen to uninfected cell antigen was higher than 2.2. 

### 2.4. Animal Experiments

ASFV-G-Δ9GL, ASFV-G-Δ9GL/ΔCD2v and ASFV-G-Δ9GL/ΔCD2v/ΔEP153R were assessed for their protective effect using 80–90 pound commercial breed swine. Groups of four pigs were inoculated intramuscularly (IM) at day 0 and 21 with 10^3^ TCID_50_ of the prescribed virus and at day 35 all animals (including a mock vaccinated control group) were IM challenged with 10^3^ TCID_50_ of highly virulent parental ASFV-G. Clinical signs (anorexia, depression, fever, purple skin discoloration, staggering gait, diarrhea and cough) and changes in body temperature were recorded daily throughout the experiment.

### 2.5. Ethics Statement

Animal experiments to collect blood for swine macrophages were performed under biosafety level 3AG conditions in the animal facilities at Plum Island Animal Disease Center (PIADC). All experimental procedures were carried out in compliance with the Animal Welfare Act (AWA), the 2011 Guide for Care and Use of Laboratory Animals, the 2002 PHS Policy for the Humane Care and Use of Laboratory Animals, and U.S. Government Principles for Utilization and Care of Vertebrate Animals Used in Testing, Research and Training (IRAC 1985), as well as specific animal protocols reviewed and approved by the PIADC Institutional Animal Care and Use Committee of the US Departments of Agriculture and Homeland Security (protocol numbers 205.03-17-R, and 225.02-19-R approved on 28 September 2017 and 9 October 2019, respectively). 

## 3. Results and Discussion

### 3.1. Development of Recombinant ASFV-G-Δ9GL/ΔCD2v and ASFV-G-Δ9GL/ΔCD2v/ΔEP153R Mutants

The effect of additional gene deletions in the genome of ASFV-G-Δ9GL was assessed by deleting two genes previously related to different aspects of ASFV virulence, CD2-like (CD2v) and C-type lectin-like (EP153R) genes. Two recombinant ASFVs were developed where the CD2v or the CD2v and EP153R genes were deleted: ASFV-G-Δ9GL/ΔCD2v and ASFV-G-Δ9GL/ΔCD2v/ΔEP153R, respectively. Deletion of the CD2v or EP153R genes from the ASFV-G-Δ9GL genome was performed by substituting most or all the amino acid residues of those ORFs with the p72GFP cassette by homologous recombination following standard methodologies [17]. Genomic modification of ASFV-G-Δ9GL/ΔCD2v results in a partial deletion of the CD2v ORF while ASFV-G-Δ9GL/ΔCD2v/ΔEP153R harbors a deletion of the CD2v and EP153R ORFs (Figure 1). ASFV-G-Δ9GL/ΔCD2v and ASFV-G-Δ9GL/ΔCD2v/ΔEP153R mutants were obtained as a pure population after successive limiting dilution purification steps in swine macrophage cell cultures.

Next generation sequencing (NGS) was used to assess both the accuracy of the genetic modifications introduced during recombination and the genomic integrity of the rest of the virus genome. Full-length genomic comparison of the recombinant ASFV-G-Δ9GL/ΔCD2v and ASFV-G-Δ9GL/ΔCD2v/ΔEP153R and parental ASFV-G-Δ9GL demonstrated a deletion of 1082 and 1629 nucleotides in ASFV-G-Δ9GL/ΔCD2v and ASFV-G-Δ9GL/ΔCD2v/ΔEP153R genomes, respectively, and of the insertion of a 1229-nucleotide construct corresponding to the p72-GFP cassette sequence. No additional significant differences were observed between these two recombinant viruses and the parental virus genomes. This confirmed that ASFV-G-Δ9GL/ΔCD2v and ASFV-G-Δ9GL/ΔCD2v/ΔEP153R did not acquire additional mutations during the process of homologous recombination or purification steps. Additionally, NGS data indicated the absence of any residual parental ASFV-G-Δ9GL genome as contaminant of the ASFV-G-Δ9GL/ΔCD2v and ASFV-G-Δ9GL/ΔCD2v/ΔEP153R stocks.

### 3.2. Assessment of the Ability of Recombinant ASFV-G-Δ9GL/ΔCD2v and ASFV-G-Δ9GL/ΔCD2v/ΔEP153R to Replicate in Swine Macrophages

To evaluate the potential effect of the deletion of CD2v and EP153R genes in the process of virus replication, the in vitro growth kinetics of recombinants ASFV-G-Δ9GL/ΔCD2v and ASFV-G-Δ9GL/ΔCD2v/ΔEP153R were assessed in swine macrophage cultures, and compared with those of the parental ASFV-G-Δ9GL in a multistep growth curve. Primary cultures of swine macrophages were infected with either recombinant ASFV-G-Δ9GL/ΔCD2v, ASFV-G-Δ9GL/ΔCD2v/ΔEP153R, parental ASFV-G-Δ9GL or field isolate ASFV-G. Infections were conducted at an MOI of 0.01 and the samples were collected at 2, 24, 48, 72, and 96 hpi.

**Figure 2:**
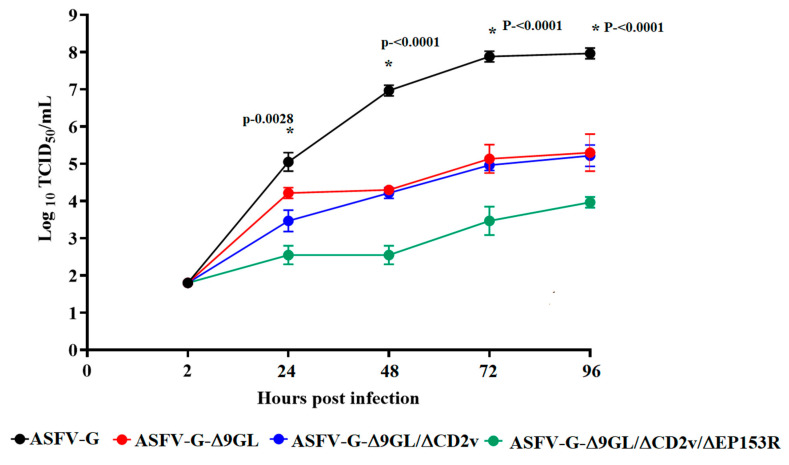
In vitro growth kinetics in primary swine macrophage cell cultures for ASFV-G-Δ9GL/ΔCD2v, ASFV-G-Δ9GL/ΔCD2v/ΔEP153R, parental ASFV-G-Δ9GL or field isolate ASFV-G. Macrophage cultures were infected (MOI = 0.01) with either virus. Samples were taken from three independent experiments at the indicated time points and titrated. Data represent the means and standard deviations. Sensitivity using this methodology for detecting virus: >log_10_ 1.8 tissue culture infectious doses (TCID_50_)/mL. Statistical significance (*) was determined using the Holm–Sidak method (α = 0.05). Computations assume that all rows are samples from populations with the same scatter (SD). Analysis was conducted on Graphpad Prism software version 8.2.1.

Results demonstrated that, as previously reported, ASFV-G-Δ9GL has a decreased replication when compared with its parental virus, the field isolate ASFV-G (Figure 2). Significant differences in virus titers (between 2.5 and 3 log_10_) were observed starting at 48 hpi until the end of the experimental period. In addition, ASFV-G-Δ9GL/ΔCD2v presented growth kinetics almost undistinguishable from that of its parental ASFV-G-Δ9GL. Conversely, ASFV-G-Δ9GL/ΔCD2v/ΔEP153R presented a clear decreased replication when compared to either its parental virus ASFV-G-Δ9GL or ASFV-G-Δ9GL/ΔCD2v. Significant titer differences (about 1–1.5 log_10_) were observed starting at 24 hpi until the end of the experiment.

While deletion of the CD2v gene does not significantly affect the ability of ASFV-G-Δ9GL to replicate in swine macrophages, the additional deletion of the EP153R gene significantly affected replication. As previously reported, the deletion of the CD2v gene does not significantly affect the replication of ASFV field isolates Malawi [18] or Georgia2010 [11]. Therefore, it is not surprising that the deletion of CD2v from ASFV-G-Δ9GL does not significantly affect virus growth in swine macrophage cultures. Regarding the EP153R gene, one wild-type (Malawi isolate) and one Vero cell-adapted (Ba71V) ASFVs were evaluated after deletion of EP153R. In both cases, the deletion of EP153R did not affect the replication of either isolate in vitro [19,20]. Interestingly, the deletion of the EP153R gene in the context of ASFV-G-Δ9GL/ΔCD2v, producing the triple deletion gene mutant ASFV-G-Δ9GL/ΔCD2v/ΔEP153R, significantly decreased the replicative abilities in swine macrophage cultures. It is not clear why the deletion of the EP153R gene affects the replication of ASFV-G-Δ9GL/ΔCD2v while it does not affect that of the Malawi or BA71V isolates [19,20]. Although swine red blood cell hemadsorption in infected cells has been shown to be solely dependent on the expression of the CD2v gene [18], it has been reported that the deletion of the EP153R gene from the BA71V genome affected CD2v expression and hemadsorption [20]. It is possible that CD2v and EP153R have differential and overlapping functions, explaining why simultaneous deletion severely affects virus replication.

### 3.3. Assessment of Recombinant ASFV-G-Δ9GL/ΔCD2v and ASFV-G-Δ9GL/ΔCD2v/ΔEP153R to Induce Protection in Immunized Pigs 

**Figure 3:**
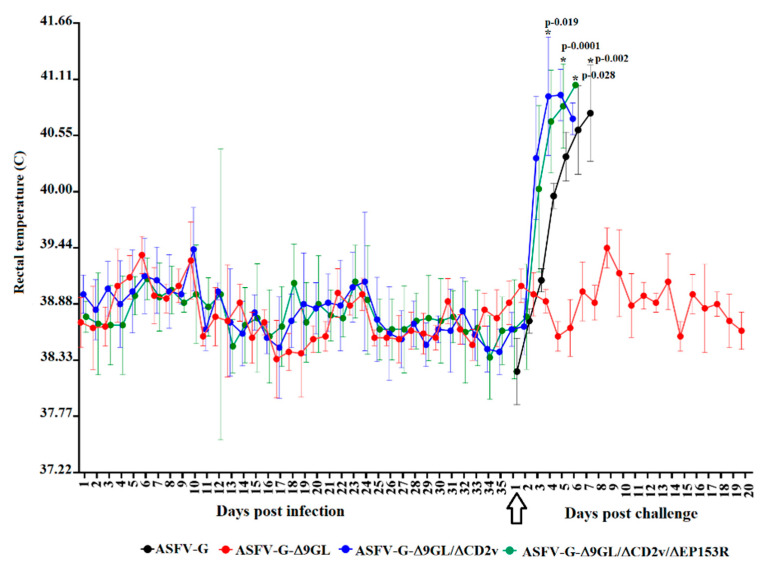
Evolution of body temperature in the animals (4 animals/group) intramuscularly (IM) infected with 10^3^ TCID_50_ of either ASFV-G-Δ9GL, ASFV-G-Δ9GL/ΔCD2v, or ASFV-G-Δ9GL/ΔCD2v/ΔEP153R. Pre- and post-challenge (indicated by the arrow) body temperatures, presented as average values and their SD, are shown for each of the experimental groups. Statistical significance (*) determined using the Holm–Sidak method (α = 0.05). Each day of sample collection was analyzed individually, without assuming a consistent SD. Analysis was conducted on Graphpad Prism software version 8.2.1.

ASFV-G-Δ9GL has been shown to be attenuated when intramuscularly (IM) inoculated in swine at doses of 10^3^ TCID_50_ and to induce a strong protective immunity when challenged with parental virulent ASFV-G isolate. The effect of the deletion of CD2v and EP153R genes in the protective efficacy of ASFV-G-Δ9GL was assessed and compared with that of the parental virus. ASFV-G-Δ9GL, ASFV-G-Δ9GL/ΔCD2v, and ASFV-G-Δ9GL/ΔCD2v/ΔEP153R were IM inoculated into three different groups (n = 4) of 80–90 pound pigs at doses of 10^3^ TCID_50_. All animals received a booster, similar route and doses, by day 21 post infection (pi). Animals in all groups remained clinically normal, including the absence of increased body temperature (>104 °F) (Figure 3).

**Figure 4:**
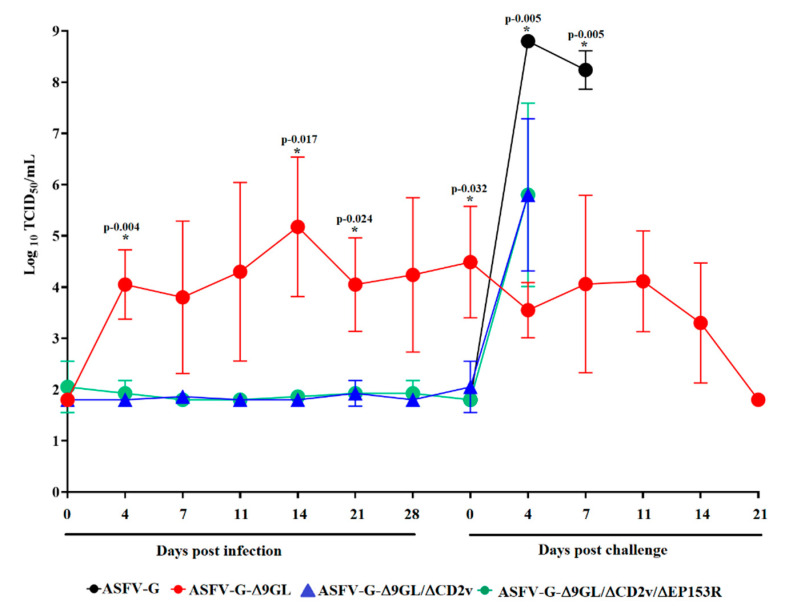
Viremia titers detected in pigs IM inoculated with 10^3^ TCID_50_ of either ASFV-G-Δ9GL, ASFV-G-Δ9GL/ΔCD2v, or ASFV-G-Δ9GL/ΔCD2v/ΔEP153R. Pre- and post-challenge viremia, presented as average TCID_50_/mL values and their SD are shown per each of the experimental groups. Sensitivity of virus detection: ≥log_10_ 1.8 TCID_50_/mL. Statistical significance (*) determined using the Holm–Sidak method (α = 0.05). Each day of sample collection was analyzed individually, without assuming a consistent SD. Analysis was conducted on Graphpad Prism software version 8.2.1.

The presence of viremia in the experimentally inoculated animals was quantified at different days post-inoculation in swine macrophage cell cultures. As expected, animals inoculated with 10^3^ TCID_50_ of virulent parental ASFV-G-Δ9GL had relatively high virus titers in blood (as high as 10^6^ TCID_50_/mL) until the day of challenge (Figure 4). Conversely, animals inoculated with mutants ASFV-G-Δ9GL/ΔCD2v or ASFV-G-Δ9GL/ΔCD2v/ΔEP153R had either very low or undetectable (test sensitivity is ≥10^1.8^ TCID_50_/mL) virus titers in blood. Therefore, it appears that the in vivo replication of both ASFV-G-Δ9GL/ΔCD2v and ASFV-G-Δ9GL/ΔCD2v/ΔEP153R was severely affected by the gene deletions. This is interesting since the deletion of CD2v from the genome of virulent isolates Georgia and Malawi [11,18] only produced a delay in the appearance of viremia and a moderate reduction in virus titers. Furthermore, the deletion of EP153R in the context of the field isolate Malawi does not have any effect on the viremia kinetics in infected animals [19]. It is interesting to note that, at least in the case of the deletion of the CD2v gene, although virus replication is not affected when evaluated in vitro using swine macrophage cultures, it is severely altered during the infection in swine, even using a route of inoculation (IM) that evades several host innate immune barriers. It is possible that these observed differences could be due to unidentified host factors that affect virus replication.

**Figure 5:**
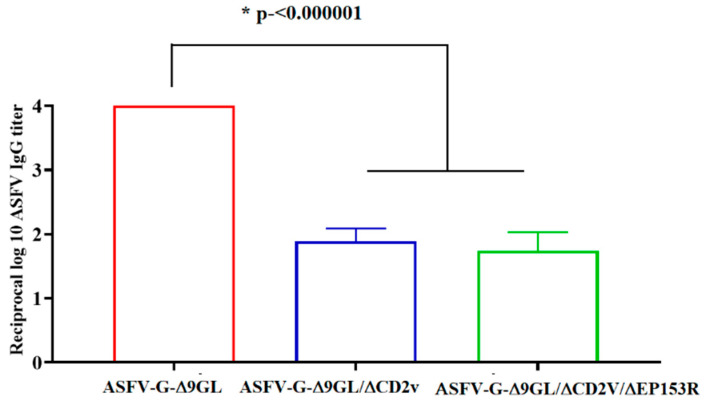
Anti-ASFV antibody titers detected in the sera of pigs IM inoculated with 10^3^ TCID_50_ of either ASFV-G-Δ9GL, ASFV-G-Δ9GL/ΔCD2v, or ASFV-G-Δ9GL/ΔCD2v/ΔEP153R by day 35 pi (day of challenge). Statistical significance (*) determined using the Holm–Sidak method (α = 0.05). Each day of sample collection was analyzed individually, without assuming a consistent SD. Analysis was conducted on Graphpad Prism software version 8.2.1.

Presence of anti-ASFV antibodies in the serum of animals inoculated with ASFV-G-Δ9GL, ASFV-G-Δ9GL/ΔCD2v, or ASFV-G-Δ9GL/ΔCD2v/ΔEP153R was detected at the time of challenge using an in-house developed direct ELISA (see Materials and Methods) (Figure 5). While animals inoculated with ASFV-G-Δ9GL presented, at the time of challenge, an average antibody titer of log_10_ 4.00 (SD ± 0.00) antibody titers in those inoculated with ASFV-G-Δ9GL/ΔCD2v or ASFV-G-Δ9GL/ΔCD2v/ΔEP153R were much lower (log_10_ 2, SD ± 0.82 and log_10_ 1.5, SD ± 0.58, respectively). As we previously reported, in our experience, the induction of virus-specific antibodies was directly related to the presence of the replication of all the attenuated viruses tested [3,4,6,7,9,10,21].

**Figure 6:**
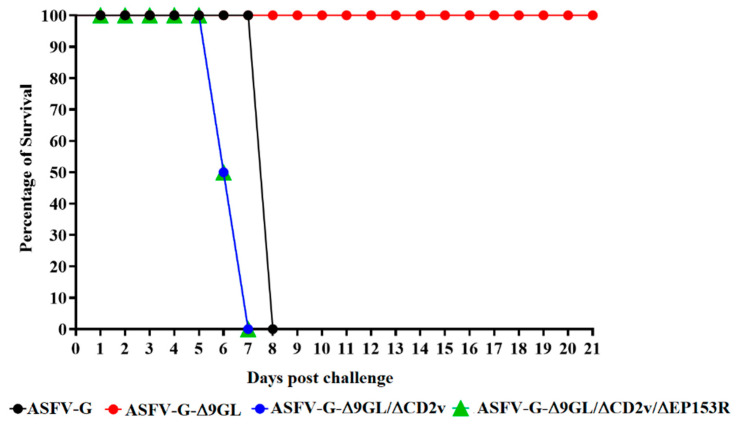
Evolution of mortality in animals (4 animals/group) IM infected with 10^3^ TCID_50_ of either ASFV-G-Δ9GL, ASFV-G-Δ9GL/ΔCD2v, or ASFV-G-Δ9GL/ΔCD2v/ΔEP153R and challenged IM with 10^3^ TCID_50_ of either ASFV-G. Comparison between the survival curves produced by groups of pigs infected with different viruses were assessed by the Log-rank (Mantel–Cox). Analysis was conducted on Graphpad Prism software version 8.2.1.

In order to assess the effect of inoculation with ASFV-G-Δ9GL/ΔCD2v or ASFV-G-Δ9GL/ΔCD2v/ΔEP153R on the induction of protection against disease, ASFV-G-Δ9GL/ΔCD2v- or ASFV-G-Δ9GL/ΔCD2v/ΔEP153R-exposed animals were challenged with parental virulent ASFV-G, with their responses compared to those infected with ASFV-G-Δ9GL. Animals previously inoculated IM at day 0 and 21 pi with 10^3^ TCID_50_ of either ASFV-G-Δ9GL, ASFV-G-Δ9GL/ΔCD2v or ASFV-G-Δ9GL/ΔCD2v/ΔEP153R were IM challenged at 35 dpi with 10^3^ TCID_50_ of virulent parental ASFV-G. Animals were monitored daily for clinical signs and changes in body temperature. In addition, four naïve animals that were challenged with parental ASFV-G using the same route and dose served as a control group. Animals inoculated with ASFV-G displayed ASF-related signs by 4–5 days post-challenge (dpc), evolving into a more severe disease in the following days and all animals being euthanized around 7 dpc (Table 1 as well as Figure 3 and Figure 6). As previously described [5], all animals receiving ASFV-G-Δ9GL survived the challenge without presenting any clinical signs related to ASF, not even a transitory rise of body temperature that is sometimes observed in protected animals by day 4–5 pc. Conversely, all animals exposed to ASFV-G-Δ9GL/ΔCD2v or ASFV-G-Δ9GL/ΔCD2v/ΔEP153R succumbed to the challenge with the parental virulent virus. Clear statistical differences were shown between the animals exposed to ASFV-G-Δ9GL and ASFV-G (*p* < 0.0002), and between ASFV-G-Δ9GL and ASFV-G-Δ9GL/ΔCD2v or ASFV-G-Δ9GL/ΔCD2v/ΔEP153R (*p* < 0.0001). The kinetics of the appearance of clinical signs, including a rise of the body temperature of ASFV-G-Δ9GL/ΔCD2v and ASFV-G-Δ9GL/ΔCD2v/ΔEP153R challenged animals, were similar to those of the control animals challenged with ASFV-G although, interestingly, significant differences arose as a result of the accelerated mortality of the pigs infected with ASFV-G-Δ9GL/ΔCD2v and ASFV-G-Δ9GL/ΔCD2v/ΔEP153R compared with the mock vaccinated group (*p*-0.0403).

Viremia kinetics after the challenge followed the presentation of clinical signs associated with disease. As expected, mock vaccinated animals presented very high virus titers in blood (as high as 10^8^–10^9^ TCID_50_/mL) until the day they were euthanized (Figure 4). Conversely, the animals exposed to ASFV-G-Δ9GL experienced a continuous decrease in their viremias until they were undetectable (test sensitivity is ≥10^1.8^ TCID_50_/mL) by the end of the experimental period. Animals exposed to ASFV-G-Δ9GL/ΔCD2v or ASFV-G-Δ9GL/ΔCD2v/ΔEP153R had a rapid increase in viremia at the only time point determined (4 dpc) since the animals were euthanized around day 5–6 pc due to the severity of the clinical signs of the disease. Therefore, it appears that the presence of pre-challenge viremia in animals inoculated with ASFV-G-Δ9GL associates with protection and an apparent limited or null replication of the challenge virus. 

In this regard, it should be mentioned that all attenuated vaccine candidates produced in our laboratory (ASFV-G-Δ9GL, ASFV-G-ΔMGF, ASFV-G-Δ9GL/ΔUK, and ASFV-G-ΔI177L strains) [3,4,5,6] which efficiently protect animals against disease after the challenge with parental ASFV-G presented, at some point before the challenge, significant viremia values. Interestingly, attenuated strains which were unsuccessful in inducing protection (as ASFV-G-Δ9GL/ΔMGF, or ASFV-G-ΔTK) [7,22] failed to induce significant viremias after their inoculation. Although the association between the induction of viremia after the administration of attenuated virus strains and protection at the challenge is clear, we cannot establish a clear cause–effect relationship between these phenomena. Host mechanisms mediating protection against ASFV infection or disease remain still largely unknown and the possible relationship between the replication of attenuated strains and the protection achieved by them should be the object of further study.

In light of the previous consideration, one possible explanation of the failure of ASFV-G-Δ9GL/ΔCD2v and ASFV-G-Δ9GL/ΔCD2v/ΔEP153R to induce protection could be the low level of replication of these viruses after administration with the possible lack of adequate antigenic exposure, resulting in a failed immune response unable to limit the replication of the challenge virus. Both CD2 and EP153R antigens have been mentioned as important in the induction of a protective immune response [23]. The host immune mechanisms responsible for protection against ASF are still under discussion. In our experience, the only immune mechanism induced by attenuated virus strains that is clearly associated with protection against challenge is the presence of virus-specific circulating antibodies [9]. In all cases, including this report, there is a strong association between the presence of ASFV-specific circulating antibodies and protection against challenge [3,4,5,9].

Genomic manipulation of ASFV seeking to improve/modify the phenotypic characteristics of a certain strain is a process of difficult predictability. It has been reported that the deletion of a specific virus gene, even those highly conserved across isolates, may produce a different phenotypic change as described for genes 9GL, TK, NL, UK, and DP148R [5,8,10,22,24,25,26,27,28]. These results suggest that the different phenotypic effects observed after deleting specific genes from two different ASFV genomes is strongly dependent on the genomic background of the viral isolate.

1. There are previous reports providing comparable data as those reported here. The deletion of a group of genes (MGF360/505) from ASFV-G-Δ9GL reduced its protective effect [7]. Similarly, Abrams et al., [29] showed that the deletion of the NL (DP71L) and UK (DP96R) genes from the genome of the naturally attenuated OUR T88/3 strain strongly reduced its ability to protect pigs against challenge with virulent virus.

It is not known what is the exact function of the AFSV CD2v and EP153R genes. CD2v has been described as responsible for mediating adhesion to swine red blood cells in the infected animals, probably facilitating the transport of the virus during infection [11,18]. In addition, the presence of CD2v produced decreased lymphoproliferative responses in swine peripheral blood mononuclear cells to specific white cell mitogens [18], although the immunomodulatory role of the protein remains to be shown. The interaction of CD2v with host protein AP-1 has been described [30] and believed to modulate vesicle transport, although the importance of that interaction during the processes of virus replication or virulence are currently unknown. Regarding EP153R, it has an inhibitory effect on the caspase-3 activation and the apoptosis induced both in ASFV-infected cells and in cell lines transfected, either stably or transiently, with the EP153R gene and is treated with different pro-apoptotic stimuli [31]. At this point, it is not clear how any of the putative functions ascribed to either CD2v or EP153R may influence the inhibitory effect induced by their deletion as described in this report.

The results presented here clearly indicate that the rational development of novel ASFV vaccines requires caution, avoiding the direct extrapolation of the results obtained by specific gene deletions obtained in a particular virus strain to novel field virus isolates. Moreover, it confirms that, although pursuing variations for specific qualities by genetically modifying ASFV is methodologically feasible, the outcome of those modifications is quite unpredictable and at each instance it will require a thorough experimental validation.

## Figures and Tables

**Table 1:** Swine survival and fever response following infection with either ASFV-G-Δ9GL, ASFV-G-Δ9GL/ΔCD2v or ASFV-G-Δ9GL/ΔCD2v/ΔEP153R and challenge with ASFV-G 10^3^ TCID_50_.

			Fever		
Virus (10^3^ TCID_50_)	No. of Survivors/Total	Mean Time to Death (±SD)	No. of Days to Onset (±SD)	Duration No. of Days (±SD)	Maximum Daily Temp, °C (±SD)
Mock	0/4	7 (0.0)	4.5 (0.58)	2.5 (0.58)	40.78 (0.47)
ASFV-G-Δ9GL	4/4	-	-	-	39.44 (0.68)
ASFV-G-Δ9GL/ΔCD2v	0/4	5.5 (0.58)	3.25 (0.5)	2.5 (0.58)	40.95 (0.26)
ASFV-G-Δ9GL/ΔCD2v/ΔEP153R	0/4	5.5 (0.58)	3.5 (0.58)	2 (0.0)	41.11 (0.38)

Animals were IM infected on day 0 and 21 pi with 10^3^ TCID_50_ of either ASFV-G-Δ9GL, ASFV-G-Δ9GL/ΔCD2v, ASFV-G-Δ9GL/ΔCD2v/ΔEP153R or mock vaccinated and challenged IM at day 35 pi with 10^3^ TCID_50_ ASFV-G.
